# Response to Therapeutic Abortion in Lactating Dairy Cows Carrying Dead Twins during the Late Embryo/Early Fetal Period

**DOI:** 10.3390/ani11092508

**Published:** 2021-08-26

**Authors:** Fernando López-Gatius

**Affiliations:** 1 Agrotecnio Centre, University of Lleida, 25198 Lleida, Spain; lopezgatiusf@gmail.com; 2 Subunit, Transfer in Bovine Reproduction SLu, 22300 Barbastro, Spain

**Keywords:** dead conceptuses, delayed luteolysis, estrus response, induced abortion failure, luteolysis failure, multiple pregnancies

## Abstract

**Simple Summary:**

The presence of dead embryos/fetuses can be precisely detected around 60 days post-insemination in dairy cattle. Little information on the fate of dead twins has been published. Practitioners usually apply a therapeutic abortion using prostaglandin F_2α_ (PGF_2α_) or its analogs to reduce the associated extended interval to estrus. This study evaluated the dose–response effect of PGF_2α_ inducing abortion in cows with two dead twins at pregnancy diagnosis 28–34 days post-AI (late embryonic period) or at confirmation of pregnancy 49–55 days post-AI (early fetal period). An increased PGF_2α_ dose had no influence on the induction of abortion and the induced abortion rate was inversely associated with milk production during the late embryonic period, whereas an increased PGF_2α_ dose was found to offer improvements during the early fetal period.

**Abstract:**

This study examined the dose–response effect of prostaglandin F_2α_ (PGF_2α_) when used to induce abortion in cows with two dead twins at pregnancy diagnosis 28–34 days post-AI (late embryonic period: LE) or at pregnancy confirmation 49–55 days post-AI (early fetal period: EF). The study population consisted of 415 cows receiving a single PGF_2α_ dose (PG1 group: 254 LE and 161 EF cows) and 200 receiving a 1.5 × PGF_2α_ dose (PG1.5 group: 121 LE and 79 EF cows). The induced abortion rate was significantly lower (chi-square test; *p* < 0.0001) in the EF (34.6%) than LE cows (88%), and was also reduced (*p* = 0.001) in the EF PG1 group (28%) compared with the EF PG1.5 group (48.1%). High milk production (≥45 kg) was the only factor influencing the induced abortion rate in LE cows (odds ratio 0.2; *p* < 0.0001), whereas the odds ratio for induced abortion of PG1.5 cows was 2.3 (*p* = 0.005) in EF cows. In conclusion, an increased PGF_2α_ dose had no effect on abortion induction during the late embryonic period and its rate was inversely associated with milk production. This higher PGF_2α_ dose did, however, offer benefits during the early fetal period.

## 1. Introduction

In cattle, pregnancy loss after its diagnosis 28–34 days post-artificial insemination (AI) mainly occurs before day 60 of gestation [1,2,3,4], when implantation is firmly established [5,6]. During this time interval covering the late embryo/early fetal period, the chances of losses are much lower [1]. The embryonic period of pregnancy runs from conception until the end of the differentiation stage (about 45 days), and the fetal period spans from day 45 of pregnancy to parturition [7]. In high producing dairy herds, the incidence of pregnancy loss can exceed 20% [2,3,4] and has a major economic impact [8]. This means that a pregnancy assessment following its diagnosis is usually scheduled for around 60 days post-AI. An important problem is that gestation markers are lost in most cows, and a cow is recorded as non-pregnant when the second diagnosis is negative. However, during this period, the presence of dead embryos/fetuses can be precisely detected, particularly in multiple pregnancies. In other words, it is very difficult to find a single dead fetus in cows carrying singletons, whereas observation of two or more dead fetuses is common. Moreover, the literature lacks information on the fate of dead twins. To reduce the extended interval to estrus associated with conceptus death, practitioners usually treat cows with prostaglandin F_2α_ (PGF_2α_) or one of its analogs. This practice is known as therapeutic abortion.

The corpus luteum (CL) of pregnancy appears essential to maintain pregnancy for the first 165 days [9] and a single PGF_2α_ dose consistently induces abortion until approximately 150 days of gestation, generally without complications [10]. However, a double PGF_2α_ dose between days 40 and 120 of gestation induces abortion in all treated cows compared with a single or lower dose, which is either less effective or totally ineffective [11]. The challenge of multiple ovulations associated with high milk production is a cogent reason to propose an increased luteolytic dose of PGF_2α_ in both pregnant and non-pregnant cows [12]. In effect, the findings of a recent study indicated that the presence of multiple corpora lutea was associated with a reduced estrus response to a single PGF_2α_ dose, whereas an increased PGF_2α_ dose was found to improve this response [13]. The objective of the present study was to assess the dose–response effect of PGF_2α_ in inducing abortion in cows with two dead twins at pregnancy diagnosis 28–34 days post-AI (late embryonic period) or at pregnancy confirmation 49–55 days post-AI (early fetal period). Possible differences in PGF_2α_ responses during the late embryonic and early fetal periods were also assessed.

## 2. Materials and Methods

### 2.1. Experimental Animals

The study population was a Holstein–Friesian dairy herd in northeastern Spain (41.13 latitude, 0.24 longitude) in which the dose–response effect of PGF_2α_ for synchronizing estrus has been established [13]. Cows were included if they were healthy as confirmed by a body condition score on a scale of 1 to 5 of 2.5–3.5 [14], produced more than 25 kg of milk per day, and were free of detectable reproductive disorders and clinical diseases during the study period (insemination days −7 to + 49–55). During this period (January 2009 to April 2016), the mean number of lactating cows in the herd was 1028 and mean annual milk production was 11,965 kg per cow. The mean annual culling rate was 29%. Cows were grouped according to age (primiparous versus pluriparous), milked three times daily, and fed complete rations.

### 2.2. Detection of Estrus, Insemination, and Pregnancy Diagnosis

Estrus was detected using a pedometer system (AfiFarm System; SAE Afikim, Israel). Walking activity values were recorded at the milking parlor (three times daily) and analyzed automatically using a herd management computer program [15]. Estrus was confirmed by palpation per rectum in cows deemed to be in estrus using the pedometer system, and the animals were inseminated at this time. Only cows with a uterus that was highly tonic and contractile to the touch, and a vaginal discharge of copious clear fluid, were inseminated. The herd was maintained on a weekly reproductive health program, as described elsewhere [13,15]. During these weekly visits, pregnancy was diagnosed at 28–34 days post-AI and confirmed at 49–55 days post-AI. All gynecological exams were performed by the author through transrectal ultrasonography. Each ovary was scanned in several planes by moving the transducer along its surface to identify luteal structures, and the number and location of corpora lutea were recorded. Scanning was also performed along the dorso/lateral surface of each uterine horn for pregnancy diagnosis. The presence of twins was established through the observation of two embryos in different positions within one uterine horn, or two embryos simultaneously present on the screen (unilateral twin pregnancy), or one embryo in each uterine horn (bilateral twin pregnancy). The viability of an embryo/fetus was confirmed by observation of a heartbeat in all exams. Only clear remnants of a dead embryo were recorded as a dead conceptus at pregnancy diagnosis 28–34 days post-AI. At this time, all cows with multiple pregnancies with at least one live embryo received a GnRH dose to promote pregnancy maintenance [16]. All procedures were approved by the Ethics Committees on Animal Experimentation of the University of Lleida.

### 2.3. Experimental Design

This experiment was designed to examine the dose–response abortion-inducing effect of PGF_2α_ in 615 cows carrying two dead twins at the time of pregnancy diagnosis 28–34 days post-AI (375 cows; late embryos: LE) or of pregnancy confirmation 49–55 days post-AI (240 cows; early fetuses: EF). In the reproductive visit, cows with LE or EF were chronologically assigned to the groups PG1 or PG1.5 according to whether they were given a single (25 mg) or 1.5 times (37.5 mg) dose of PGF_2α_ (dinoprost i.m.; Enzaprost, CEVA Santé Animale, Barcelona, Spain). A total of 415 cows were assigned to the PG1 group (254 LE and 161 EF cows) and 200 cows were included in the PG1.5 group (121 LE and 79 EF cows). Cows showing estrus within seven days after treatment were either inseminated (LE cows) or not inseminated and estrus was recorded (EF cows). During the early fetal period, cows were not inseminated to assess the complete expulsion of gestation remains the next visit. Luteal structures and uterine contents of non-inseminated LE cows and of all EF cows were assessed seven days after treatment. Bilateral multiple pregnancies detected during the early fetal period were excluded, as in this herd, twin reduction by amnion rupture [17,18] is routinely performed in all bilateral pregnancies.

### 2.4. Data Collection and Statistical Analyses

The induced abortion rate was defined as the percentage of cows showing estrus out of the total number of cows in each treatment group during the seven days following treatment and/or absence of CL seven days after treatment. The following data were recorded in each animal: parturition and treatment dates; lactation number (parity, primiparous vs. pluriparous); number and site of conceptuses (unilateral vs. bilateral in LE cows, and right vs. left in unilateral pregnancies); treatment (PG1 or PG1.5); milk production at treatment (mean production in the three days before treatment) (low producers < 45 kg vs. high producers ≥ 45 kg); estrus response to PGF_2α_ treatment (presence vs. absence); days from treatment to estrus (continuous); and pregnancy 28–34 days post-AI for inseminated LE cows. The threshold for milk production was set as the median value of production recorded in primiparous cows. Treatment dates were used to assess the effects of season on subsequent reproductive performance. In our geographical region, there are only two clearly differentiated weather periods: warm (May to September) and cool (October to April) [19,20].

Overall reproductive performance in the two treatment groups was compared using the chi-square test (percentages). The effects of treatment on the interval (days) from PGF_2α_ treatment to estrus were assessed by ANOVA and Tukey post hoc tests.

The effects of treatment on the estrus response and induced abortion rates were analyzed by binary logistic regression. Two regression analyses were performed using estrus response and abortion after PGF_2α_ treatment as the dependent variables for both LE and EF cows. The factors entered in the models as independent variables were treatment, conceptus site (unilateral vs. bilateral in LE and right vs. left in EF pregnancies), year and season of treatment, parity, and milk production at treatment. Possible interactions between treatment and the variables milk production, season, and parity were also examined. Regression analyses were conducted according to the method of Hosmer and Lemeshow [21] using the logistic procedure of PASW Statistics for Windows Version 18.0 (SPSS Inc., Chicago, IL, USA). Significance was set at *p* < 0.05. Values are expressed as the mean ± standard deviation (S.D.).

## 3. Results

**Figure 1:**
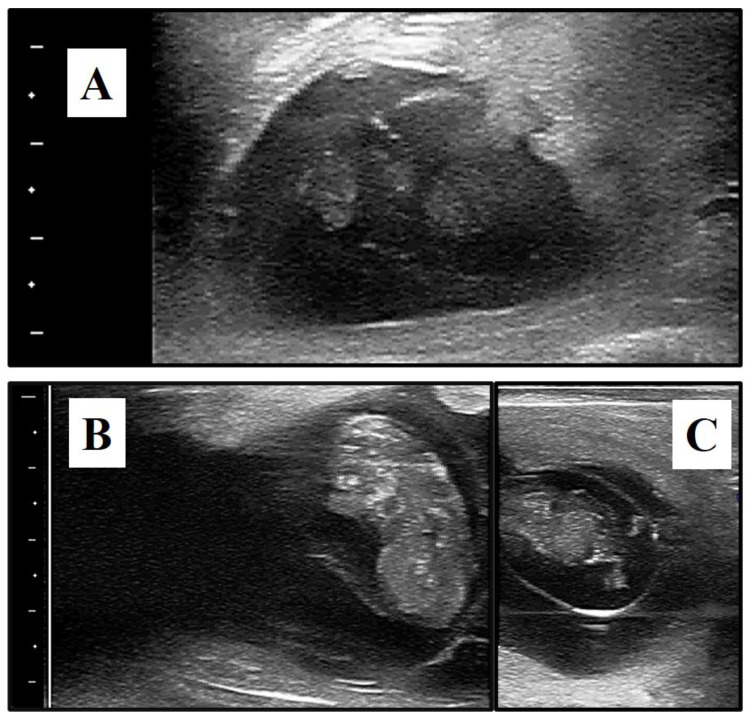
Sonograms showing signs of degeneration in both twins and turbidity of the uterine content 34 days post-AI (**A**) or one fetus seemingly of normal size and development (**B**) alongside its partner showing signs of deterioration such as floating hind limbs within the amnion at 52 days post-AI (**C**). Bar spacing: 10 mm.

Mean milk production and days in milk at the time of treatment, as well as the number of lactations, were 48 ± 10 kg (26.2–81.4 kg), 141.5 ± 63.1 days (81–538 days), and 2.8 ± 1.7 lactations (1–9 lactations), respectively (mean ± SD, range in parentheses). In 358 (95.5%) of the 375 pregnancies found to carry dead twins at 28–34 days post-AI, the embryos were located in the same uterine horn (243 (67.9%) right and 115 (32.1%) left). The remaining 17 (4.5%) were bilateral twin pregnancies. Triplets and quadruplets were not recorded. Among the 240 pregnancies carrying dead twins at 49–55 days post-AI, the twins were located in the right uterine horn in 152 (63.3%) and the left uterine horn in 88 (36.7%). Co-twin degeneration, turbidity, and formation of floating debris were observed in most pregnancies at 28–34 days post-AI (Figure 1a), whereas at 49–55 days post-AI, one fetus that seemed normal in size and development (Figure 1b) was detected alongside its partner, which showed signs of degeneration (Figure 1c).

The independent variables recorded in each of the two treatment groups and effects of the different treatments on the induced abortion rate for both LE and EF cows are shown in Table 1. According to chi-squared tests, the LE group showed a significantly (*p* < 0.0001) lower proportion of pluriparous cows (64.3%) and of cows treated during the warm season (21.6%) compared with the group of EF cows (94.6% and 47.5%, respectively). Estrus was detected in 285 LE cows (76%), all of which were inseminated, and 121 (42.5%) became pregnant following AI. The estrus response rate was significantly lower (*p* < 0.0001) in the EF cows (27.9%) and significantly reduced (*p* = 0.007) in the EF PG1 (22.4%) group compared with the EF PG1.5 group (39.2%). Estrus was detected significantly (ANOVA and Tukey post-hoc test: *p* < 0.0001) earlier in LE cows (*n* = 285; 2.8 ± 0.9 days) than in EF cows (*n* = 67; 5.6 ± 0.9 days). The induced abortion rate was significantly lower (*p* < 0.0001) in the EF cows (34.6%) compared with the LE cows (88%), and in the EF PG1 cows (28%) compared with the EF PG1.5 cows (48.1%) (*p* = 0.001). All non-aborting cows preserved their luteal structures and uterine contents seven days after treatment.

Based on binary logistic regression procedures, the odds ratio for an estrous response was 0.2 (95% confidence interval: 0.1–0.3; *p* < 0.0001) during the warm period of the year in LE cows: 42/81 (51.9%) versus 243/294 (82.7%). Treatment, year, parity, milk production, and conceptus site were not included in the final model. Treatment–season, treatment–parity, or treatment–milk yield interactions were not found. No factors were noted to affect the estrus response in EF cows.

Table 2 provides induced abortion rates, odds ratios, and 95% confidence intervals for LE and EF cows. For LE cows, the final model included the effect of milk production as the only factor affecting the induced abortion rate with an odds ratio of 0.2 (*p* < 0.0001) for high-producing cows (≥45 kg). For EF cows, the final model included the effect of the warm period of the year and treatment. Year, parity, milk production, and conceptus site were not found to be significant and were not included in the final model. No treatment–season, treatment–parity, or treatment–milk yield interactions were found. The odds ratio for induced abortion was 8.9 (*p* < 0.0001) during the warm period. Taking PG1 cows as reference, the odds ratio for induced abortion of PG1.5 cows was 2.3 (*p* = 0.005).

## 4. Discussion

As far as we know, no previous study has examined the impacts of an increased PGF_2α_ dose on induced abortion in lactating dairy cows carrying dead twins during the late embryo or early fetal period. The points to be highlighted are as follows: (1) during the late embryonic period, an increased PGF_2α_ dose had no effect on abortion induction, induced abortion had no effect on subsequent fertility, and the induced abortion rate was inversely related to milk production; (2) during the early fetal period, the 1.5 PGF_2α_ dose led to a higher induced abortion rate compared with the single PGF_2α_ dose, particularly during the warm period, and this rate was significantly lower than that recorded for the late embryonic period; and (3) cow-specific differences such as proportions of pluriparous cows and of cows treated during the warm period were found between the two development periods.

During the late embryonic period, an increased PGF_2α_ dose had no beneficial effects in cows carrying dead twins, whereas the estrous response, treatment to estrus interval, and subsequent conception rate were similar to those recorded during the same period in the same herd in cows with a single CL receiving a single PGF_2α_ dose [13]. This quick response to PGF_2α_ treatment irrespective of dose could be explained by the gradual dissolution of conceptuses (Figure 1a) involving the influx and migration of large populations of white blood cells, local release of cytokines, and possible release of prostaglandins from uterine tissues. It may be thus concluded that PGF_2α_-induced abortion during the late embryonic period was not detrimental to fertility. However, it is harder to understand why high producers were less responsive to PGF_2α_ treatment during this period. A very significantly (*p* < 0.0001) reduced abortion rate was observed in cows producing more milk, reinforcing previous results in which the presence of multiple CL was found to reduce the luteolytic response to a single PGF_2α_ dose synchronizing estrus in high milk producers [13]. Metabolic stress in high-producing dairy cows could be expressed as an abnormal endocrine balance, thus jeopardizing complete luteolysis and the subsequent estrus response. The higher metabolic clearance of steroid hormones linked to high milk production [22,23,24] could lead to a prolonged period of low progesterone and, in turn, to the reduced sensitivity of CL to lysis by PGF_2α_ [10].

The 1.5-times PGF_2α_ dose proposed here was beneficial for inducing abortion during the early fetal period, with an odds ratio of 2.3 (*p* = 0.005). However, only 48% of cows in this group aborted, far less than the 88% recorded during the late embryonic period. The fact that estrus appeared significantly (*p* < 0.0001) earlier in the LE (2.8 days) than EF (5.6 days) cows suggests that luteolysis was delayed in the latter. When a single PGF_2α_ dose was administered during the early fetal period in cows with one single CL, complete luteolysis occurred in 100% of cows only 14 days after treatment [11]. It is likely that, by prolonging the time after treatment to control luteolysis, the induced abortion rate in the present study would have been higher. While the induced abortion rate increased significantly (*p* < 0.0001) during the warm period, probably because of compromised luteal function due to heat stress [25]. A main question that arises is the manner in which the presence of dead twins seems to prolong luteal activity, irrespective of treatment. In single pregnancies suffering spontaneous embryo death, conceptus expulsion more than resorption has been reported [26]. However, a cow bearing twins, one of which is dead, is unable to selectively expel the individual conceptus [27,28]. When dead twins are detected during the early fetal period, it is common to find one normal fetus in terms of size and development (Figure 1b) alongside its degenerated twin (Figure 1c). This suggests that one fetus “protects” the other for a period of time until pregnancy loss or maintenance of a single pregnancy [27]. In these cases, a fused placenta larger than in a single pregnancy may be present. The incidence of placental anastomosis exceeds 90% in cattle [29] and the longer interval to estrus or luteolysis failure after treatment may be explained by survival of trophoblastic cells. Intrauterine infusion of embryonic homogenates [30] or trophoblast proteins [31,32] have been shown to favor luteotropic signals and extend luteal function. From a practical perspective, our results suggest benefits of an increased PGF_2α_ dose, probably a double dose or more, in cows carrying dead twins during the early fetal period.

Cow-specific differences between our LE and EF groups were expected. Thus, a greater proportion of primiparous cows (35.7%) were diagnosed with dead twins during the late embryonic period than during the early fetal period (5.4%). Mechanical competition between twins could favor earlier development failure in younger cows with a smaller uterus than pluriparous cows. The fact that the warm season, a strong factor impairing pregnancy in our geographical area [33], had no influence on the induced abortion rate reinforces this assertion.

A further expected result was the low incidence (4.5%) of bilateral dead twins during the late embryonic period. Under good management and environmental conditions, the ratio between unilateral and bilateral multiple pregnancies is normally close to one [34]. Here, the ratio was 21.2 (95.5/4.5), indicating a clear predominance of dead unilateral twins upon the first pregnancy diagnosis 28–34 days post-AI. The risk of pregnancy loss during the first trimester of gestation in cows carrying live twins may be from five to nine times higher for unilateral than bilateral twins [27,35].

## 5. Conclusions

A higher than normal PGF_2α_ dose given during the late embryonic period had no effect on abortion induction and the rate of this factor was found inversely related to milk production. In contrast, an increased PGF_2α_ dose was found to be beneficial for inducing abortion in the early fetal period. As the induced abortion rate in this later period (48%) was much lower than in the late embryonic period (88%), we would recommend the use of an increased PGF_2α_ dose in cows carrying dead twins during this early fetal period.

## Figures and Tables

**Table 1:** Independent variables recorded at the time of treatment and effects of the different treatments on each dependent variable (*n* = 615).

Treatment ^(a)^	LE PG1 (*n* = 254)	LE PG1.5 (*n* = 121)	Total LE (*n* = 375)	EF PG1 (*n* = 161)	EF PG1.5 (*n* = 79)	Total EF (*n* = 240)
Independent variables ^(b)^						
Parity (pluriparous)	158 (62.2%) *	83 (68.6%) *	241 (64.3%) *	151 (93.8%) **	76 (96.2%) **	227 (94.6%) **
Milk production (≥45 kg)	130 (51.2%)	62 (51.2%)	192 (51.2%)	79 (49.1%)	39 (49.4%)	118 (49.2%)
Season (warm period: May–September)	54 (21.3%) *	27 (22.3%) *	81 (21.6%) *	74 (46%) **	40 (50.6%) **	114 (47.5%) **
Bilateral twins	12 (4.7%)	5 (4.1%)	17 (4.5%)			
Unilateral twins (right uterine horn)	164 (64.6%)	79 (65.3%)	243 (64.8%)	101 (62.7%)	51 (64.6)	152 (63.3%)
Dependent variables ^(c)^						
Estrus ^(d)^	194/254 (76.4%) *	91/121 (75.2%) *	285/375 (76%) *	36/161 (22.4%) **	31/79 (39.2%) ***	67/240 (27.9%) **
Days (mean ± SD) ^(e)^	2.8 ± 0.9 *	2.7 ± 0.9 *	2.8 ± 0.9 *	5.5 ± 0.9 **	5.8 ± 0.9 **	5.6 ± 0.9 **
Conception rate ^(f)^	83/194 (42.8%)	38/91 (41.8%)	121/285 (42.5%)			
Abortion ^(d)^	221/254 (87%) *	109/121 (90.1%) *	330/375 (88%) *	45/161 (28%) **	38/79 (48.1%) ****	83/240 (34.6%) **

^(a)^ LE PG1: cows with late embryos (28–34 days post-AI) receiving a single PGF_2α_ dose (25 mg dinoprost); LE PG1.5: cows with late embryos receiving a 1.5 × PGF_2α_ dose (37.5 mg dinoprost); EF PG1: cows with early fetuses (49–55 days post-AI) receiving a single PGF_2α_ dose; EF PG1.5 cows with early fetuses receiving a 1.5 × PGF_2α_ dose. All LE cows showing estrus were inseminated. ^(b)^ Values with different superscripts within rows denote significant differences detected by the chi-square test (*^-^**: *p* < 0.0001). ^(c)^ Values with different superscripts within rows denote significant differences detected by the chi-square test (percentages) or ANOVA and Tukey post-hoc tests (means ± SD). ^(d)^ In all cows, *^-^**^,^ *^-^***^,^ *^-^****: *p* < 0.0001; **^-^***: *p* = 0.007; **^-^****: *p* = 0.001. ^(e)^ Days from PGF_2α_ treatment to estrus ranging from 1 to 7 days (LE) and from 3 to 7 days (EF) (*^-^**: *p* < 0.0001). ^(f)^ In inseminated cows.

**Table 2:** Odds ratios for induced abortion rate of the variables entered in the final logistic regression model for cows with late embryos (*n* = 375) and cows with early fetuses (*n* = 240).

Factor	Class	*n*	% Induced Abortion	Odds Ratio	95% Confidence Interval	*p*
LE cows ^(a)^						
Milk production	<45 kg	168/183	91.8	Reference		
	≥45 kg	142/192	74	0.2	0.09–0.5	<0.0001
EF cows ^(b)^						
Season ^(c)^	Cool	30/126	23.8	Reference		
	Warm	53/114	46.5	8.9	2.7–28.7	<0.0001
Treatment ^(d)^	PG1	45/161	28	Reference		
	PG1.5	38/79	48.1	2.3	1.3–4.3	0.005

^(a)^ LE cows: cows with late embryos (28–34 days post-AI). Hosmer and Lemeshow goodness-of-fit = 22.1; 3 df; P = 0.91. R^2^ Nagelkerke = 0.12. ^(b)^ EF cows: cows with early fetuses (49–55 days post-AI). Hosmer and Lemeshow goodness-of-fit = 25.6; 3 df; P = 0.92. R^2^ Nagelkerke = 0.20. ^(c)^ Cool period: October–April; warm period: May–September. ^(d)^ PG1: cows receiving a single PGF_2α_ dose (25 mg dinoprost); PG1.5: cows receiving a 1.5 × PGF_2α_ dose (37.5 mg dinoprost).

## Data Availability

The data presented in this study are available on request. These data are not publicly available to preserve the data privacy of the commercial farm.
